# Prophylactic effect of omega-3 polyunsaturated fatty acids monotherapy in preventing recurrent major depressive disorder: A randomized controlled trial

**DOI:** 10.37796/2211-8039.1667

**Published:** 2025-12-01

**Authors:** Ikbal Andrian Malau, Cheng-Ho Chang, Wei-Jen Chen, Wen-Chun Liu, Halliru Zailani, Hsien-Feng Liao, Jane Pei-Chen Chang, Wei-Che Chiu, Kuan-Pin Su

**Affiliations:** a Mind-Body Interface Research Center (MBI-Lab) and Department of Psychiatry, China Medical University Hospital, Taichung, Taiwan; b Graduate Institute of Biomedical Sciences, College of Medicine, China Medical University, Taichung, Taiwan; c Department of Psychiatry, Kaohsiung Veterans General Hospital, Kaohsiung, Taiwan; d An Nan Hospital, China Medical University, (CMU) Tainan, Taiwan; e Department of Nursing, National Tainan Junior College of Nursing, Tainan, Taiwan; f Graduate Institute of Nutrition, China Medical University, Taichung, Taiwan; g Department of Biochemistry, Ahmadu Bello University, Zaria, Nigeria; h Jenteh Junior College of Medicine, Nursing and Management, Miaoli, Taiwan; i College of Medicine, China Medical University, (CMU) Taichung, Taiwan; j Child and Adolescent Psychiatry Division, China Medical University Hospital, Taichung, Taiwan; k Department of Psychiatry, Cathay General Hospital, Taipei, Taiwan; l School of Medicine, Fu Jen Catholic University, Taipei, Taiwan; m Mind-Body Interface Research Center (MBI Lab & Care), CMU Hospital, Taichung, Taiwan; n Office of Research and Development, Asia University, Taichung, Taiwan

**Keywords:** Major depressive disorder, n-3 PUFAs, Recurrence, Monotherapy, Prophylactic

## Abstract

**Background:**

Omega-3 polyunsaturated fatty acids (n-3 PUFAs) have demonstrated efficacy as adjunctive treatment for MDD. In fact, fewer studies assessed the prophylactic properties of n-3 PUFAs as monotherapy on the recurrence of MDD.

**Aims:**

This study aimed to assess the prophylactic effect of n-3 PUFAs monotherapy against recurrent MDD.

**Methods:**

We conducted a 6-month randomized controlled trial to assess the prophylactic effect of n-3 in preventing recurrent MDD. We assigned 60 remitted MDD patients to the n-3 group (n = 30) and placebo (n = 30). Furthermore, we assessed the difference in depression severity and MDD recurrence based on the 21-item Hamilton Rating Scale for Depression (HRSD) at months 1, 2, 3, 4, and 6 between groups. The recurrent event of MDD was defined as an HRSD score >20. Furthermore, biochemical parameters in plasma were assessed as the secondary outcomes.

**Results:**

There was no significant difference in the HRSD score between the n-3 group and placebo each month ( *p-*value >0.05). However, our findings have implicated that omega-3 monotherapy for MDD contributed to a lower recurrence rate compared to the placebo group at month 6 ( *p*-value = 0.035). Omega-3 supplementation was superior to placebo to preventing recurrent MDD analyzed using Kaplan–Meier survival analysis over a 6-month study period ( *p-*value = 0.041). In comparison, the eicosapentaenoic acid (EPA) plasma level of the n-3 group at the end point of study was significantly higher than the placebo ( *p*-value = 0.023), but not for docosahexaenoic acid (DHA) ( *p*-value = 0.119).

**Conclusion:**

Our study concluded that n-3 PUFAs monotherapy demonstrated a prophylactic effect on the recurrence of MDD.

## 1. Introduction

Major depressive disorder (MDD) is one of the leading causes of disability worldwide compared to other mental disorders [1] and is associated with multiple detrimental outcomes [2,3]. Persons with a history of MDD are at risk of experiencing a higher level of recurrence [4]. Moreover, MDD is challenging as the standard treatments often result in treatment-resistant depression followed by mild to severe adverse effects [5]. Given these complexities, omega-3 polyunsaturated fatty acids (n-3 PUFAs) are considered a potential alternative treatment for various psychiatric disorders [6–8]. Additionally, n-3 PUFAs have been studied for their potential benefits in managing MDD [9–11]. Hence, clinical evidence reveals a correlation between lower plasma n-3 PUFAs levels and an increased risk of depression [12]. N-3 PUFAs supplementation elevated plasma levels in MDD patients with higher baseline n-3 PUFA levels, leading to remission. Conversely, patients with lower baseline n-3 PUFA levels showed non-significant plasma level increases and ongoing depressive symptoms [13]. Therefore, the necessary dose and treatment duration might vary based on the individual’s baseline omega-3 fatty acid levels.

As an essential nutrient, n-3 PUFAs cannot be synthesized in the human body; therefore, it is crucial to emphasize the importance of n-3 PUFA-enriched diet and supplementation [14,15]. A prior network meta-analysis of ten clinical studies published in 2020 demonstrated that high-dose (≥2 g) n-3 PUFAs supplementation was associated with greater reductions in mood scores compared to low-dose (<2 g) [16] and well tolerated [17]. Highly concentrated EPA and DHA are the main components in n-3 PUFAs that have shown beneficial effect in mood disorders [18]. Particularly, supplementation with EPA-predominant n-3 PUFAs formulation is found to possess stronger potential for anti-inflammatory effects in depression [19]. The recommended ratio of eicosapentaenoic acid (EPA) and docosahexaenoic acid (DHA) (>2:1) is considered effective in promoting antidepressant effect on MDD [20]. Therefore, supplementing n-3 PUFAs in dietary intake must be in accordance with the ratio of higher EPA than DHA for people with depression to reach the beneficial effects [20]. Nevertheless, it’s important to highlight that the precise effective dose of n-3 PUFAs for treating MDD remains unclear due to the mixed findings [19].

Several clinical trials were conducted to evaluate the therapeutic efficacy of n-3 PUFAs in MDD. However, the majority of studies have explored the adjuvant use of n-3 PUFAs in combination with antidepressants that contributed to lower mood scores [21,22]. Conversely, there are only a few studies considered n-3 PUFAs as monotherapy to treat MDD. Two studies highlighted the efficacy of DHA monotherapy [23,24], while another study reported beneficial effects of EPA monotherapy [25] in improving depressive symptoms among MDD patients. In addition, there is a study published in 2020 provided insight that EPA-predominant n-3 PUFAs monotherapy contributed to lowered mood scores in patients with cardiovascular diseases (CVD) comorbid MDD [26]. However, there has been no study evaluating the prophylactic effect of EPA-predominant n-3 PUFAs as monotherapy on MDD recurrence in remitted patients. Furthermore, this multi-site randomized controlled trial (RCT) aimed to fill this research gap by examining the potential of n-3 PUFAs monotherapy in preventing the recurrence of MDD, independent of antidepressant medications in remitted MDD patients. By investigating the prophylactic effects of n-3 PUFAs monotherapy, this study will provide significant insights into the role of nutritional interventions in maintaining remission and preventing the recurrence of depressive episodes in persons with a history of MDD.

## 2. Methods

### 2.1. Subjects and study design

We conducted a 6-month multi-center randomized controlled trial to assess the effectiveness of n-3 PUFAs monotherapy in preventing the recurrence of MDD. We recruited eligible participants from three outpatient psychiatry departments from different hospitals (China Medical University Hospital and Taipei Cathay Hospital) and the study adhered to the ethical principles laid out in the 1964 Declaration of Helsinki and its subsequent amendments.

The inclusion criteria for the study were: participants aged 18–65 years who had met the Diagnostic and Statistical Manual of Mental Disorders, Fourth Edition (DSM-IV) diagnosis of major depressive disorder in the past year and were currently in full remission, having two or fewer depressive symptoms in the last eight weeks, and a 21-item Hamilton Rating Scale for Depression (HRSD) score of less than 7 (non-depressive symptom) at the baseline. In addition, we ensured that the depression levels of all participants at baseline were classified as mild or less by using the Beck Depression Inventory (BDI) questionnaire, which was not greater than 18. The HRSD is a clinician-administered scale that objectively evaluates the severity of depression, focusing on somatic and physical symptoms. In contrast, the BDI is a self-reported questionnaire that captures patients’ subjective experiences, emphasizing emotional and cognitive aspects of depression. Using both scales provides a well-rounded assessment by integrating objective clinical observations with the patient’s personal perspective. All participants in this study did not receive antidepressants or psychosocial therapies during the study and were required to have a full understanding of the entire research plan before signing an informed consent. Participants who were diagnosed with schizophrenia, bipolar disorder, psychotic disorder, organic mental disorder, substance use disorder, acute psychotic state or strong suicidal intention, and allergy to fish oils were excluded from the study. Institutional review board (IRB) approvals were obtained from China Medical University Hospital (DMR93-IRB-87) and Taipei Cathay Hospital (CT9336). This trial was registered on the ClinicalTrials.gov with identifier: NCT00816322.

### 2.2. Randomization

Sixty eligible patients were included and randomly assigned to n-3 group and placebo, in a 1:1 ratio using computer-generated randomization with block randomization. An independent researcher performed the randomization, and the allocation sequence remained unknown to the research team until the databases were finalized.

### 2.3. Interventions

**Fig. 1:**
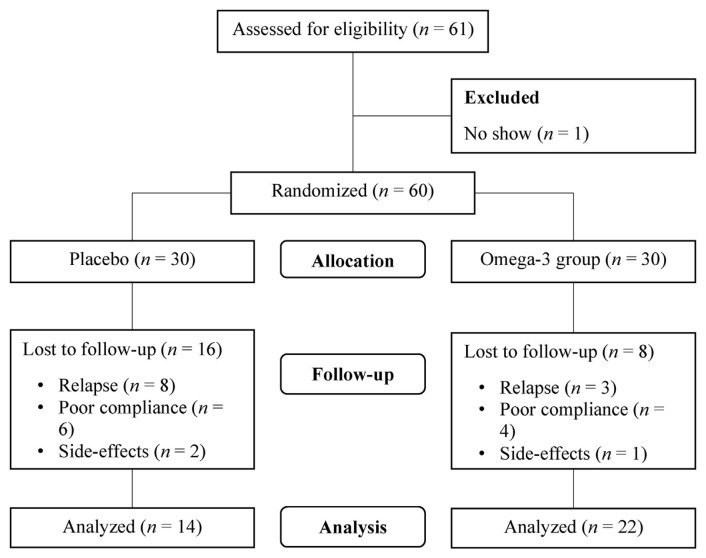
Flow diagram of the progress through the phases of the clinical trial and present analysis.

Intervention was given to n-3 group with a fish oil capsule of EPA and DHA (EPA 2.1 g, DHA 1.1 g) and the source of n-3 fatty acids is menhaden fish, while placebo group was provided with a high oleic oil capsule. Each subject in both groups consumed 4 capsules per day. The placebo group was administered an equal number of placebo capsules as the n-3 group for 6 months, and the physical characteristics such as appearance and weight of each capsule will be indistinguishable between the two groups. To minimize any potential bias, both the fish oil and placebo capsules were packaged to be odorless and had a similar flavor. This ensured masking participants from intervention. The cessation of treatment in the study was prompted by several reasons including recurrence, intention or risk of suicide (HRSD-21 suicide item score ≧3), condition requiring hospitalization, subjective decision to discontinue treatment, and discontinuation of treatment due to side effects. In the n-3 group, eight patients decided to discontinue the study, with three experienced MDD relapse with unstable emotion and prescribed with medication, four had poor compliance with irregular visit and did not show up, and one patient requested to stop due to mild side-effects such as unable to swallow the capsules. On the other hand, sixteen patients in the placebo group discontinued the study, of whom eight experienced MDD relapse and were prescribed with medication, six had poor compliance with irregular visit and did not show up, and two requested to stop due to mild side-effects such as an allergy and unable to swallow the capsules. The details of the subjects’ recruitment, allocation, and analysis are presented in Fig. 1.

### 2.4. Clinical assessment

This study used the 21-item HRSD scale as a reliable diagnostic tool to measure depression levels. The clinical team conducted interviews with participants using a semi-structured questionnaire and calculated the total score of the 21-item HRSD scale to assess the level of depression in all participants at month 1, 2, 3, 4, 5, and 6. Thus, further analysis of the incidence of depressive symptoms was assessed both in the n-3 group and placebo at each time point. Recurrent events of MDD were evaluated using an HRSD score greater than 20. Trained clinical nurses were masked and assigned for the clinical assessment.

### 2.5. Biochemical parameters

As part of the study, venous blood samples were collected for biochemical parameters analysis. Gas chromatography, a powerful and precise technique, was used to analyze the relative amounts fatty of acids in blood samples. The fatty acid compositions of the patients were analyzed using gas chromatography with fatty acid methyl esters (FAMEs). By comparing the retention times of the unidentified fatty acids with suitable standard FAME, their compositions were determined. Fatty acid levels were assessed using red blood cells (RBCs) because they can reflect long-term fatty acid status. The process involves isolating RBCs, extracting lipids, converting fatty acids to FAMEs through transesterification, and analyzing them using gas chromatography. Gas chromatography separates and quantifies fatty acids with results interpreted using reference standards. The protocol with detailed scheme was explained in previous study [27]. Subsequently, we compared erythrocyte fatty acid levels between two arm groups at baseline and endpoint (month 6) of study. This study also assessed the changes in major biochemical parameters related to the side effects of intervention on liver, kidneys, hearts, blood clotting, blood glucose. Thus, we assessed the levels of aspartate aminotransferase (AST/GOT), alanine aminotransferase (ALT/GPT), blood urea nitrogen (BUN), creatinine, cholesterol, triglycerides, low-density lipoprotein (LDL) cholesterol and high-density lipoprotein (HDL) cholesterol, prothrombin time (PT), activated partial thromboplastin time (aPTT), glucose, and prolactin. The biochemical parameters were assessed using standardized methods. AST, ALT, BUN, creatinine, cholesterol, triglycerides, LDL, HDL, and glucose were measured using enzymatic or colorimetric assays on automated biochemistry analyzers (e.g., Roche Cobas). PT and aPTT were evaluated through coagulation assays on coagulation analyzers (e.g., Sysmex CA-series). Prolactin levels were determined using ELISA. Serum samples were used for most parameters, while plasma was used for PT and aPTT. All subject data were kept confidential, and the examiner remained blinded to ensure intervention accuracy.

### 2.6. Sample size estimation

In this study, we expected that the intervention would lead to a decrease of 2 points in the HRSD (Hamilton Rating Scale for Depression) score. The population standard deviation is estimated to be 3 according to previous similar RCT study, indicating the variability of HRSD scores within the population. The study follows a balanced design with an equal number of participants assigned to both the control and intervention groups. The objective is to compare the mean HRSD scores between the two groups to evaluate the impact of the intervention on depression levels. To estimate the sample size, we assumed α = 0.05 and power = 0.80 (β = 0.20, therefore 1-β = 0.8). Initially, the number of patients in each group was set to 16, but to account for potential dropouts, we increased the sample size to 30 in each group.


$$n=\frac{2\left[{\left(a+b\right)}^{2}\sigma^{2}\right]}{{\left(\mu_{1}-\mu_{2}\right)}^{2}}$$

Where *n* is sample size in each group, *μ_1_-μ_2_* is population mean in treatment group-population mean in placebo, *σ^2^* is population variance (SD), *a* is conventional multiplier for alpha* when alpha is 0.05, and *b* is conventional multiplier for power* when beta is 0.80.

### 2.7. Statistical analysis

We performed all statistical analysis using SPSS v.26 software (SPSS Inc., Chicago, IL, USA). The HRSD assessments before and after treatment were collected. The difference in characteristics and severity of depression at baseline and the HRSD scores at each month assessments between groups were compared by Mann–Whitney U-test. Chi-squared (χ2) test was used to compare the frequency of recurrences between two groups. Kaplan–Meier Survival analysis was used to evaluate survival function, and log-rank test to compare the difference survival rate in recurrence of MDD between two groups as the primary outcomes. We analyzed the comparison of erythrocyte PUFAs levels and other biochemical parameters between two groups at baseline and month 6 (endpoint) as the secondary outcomes using Mann–Whitney U-test. The significance of the difference was set at *p*-value< 0.05.

## 3. Results

### 3.1. Characteristics of participants at baseline

This study ensured that there were no differences in characteristics of subjects between placebo and the n-3 group at baseline. Table 1 shows from 60 subjects that assigned to two groups, there were no differences in age ( *p*-value = 0.765) and sex ratio ( *p-*value = 0.994) at baseline. Moreover, depression severity that assessed using both HRSD and BDI did not differ between the n-3 group and placebo group ( *p*-value> 0.05).

### 3.2. Comparison of HRSD scores between two groups at each time point

Table 2a shows that there is no significant difference in the HRSD mean score between groups at each time point of the study period ( *p*-value> 0.05). However, HRSD mean score was lower in the n-3 group than in placebo group at each time. Table 2b shows MDD patients in the n-3 group had a lower recurrence rate compared to the placebo group at month 6 ( *p*-value = 0.035).

**Fig. 2:**
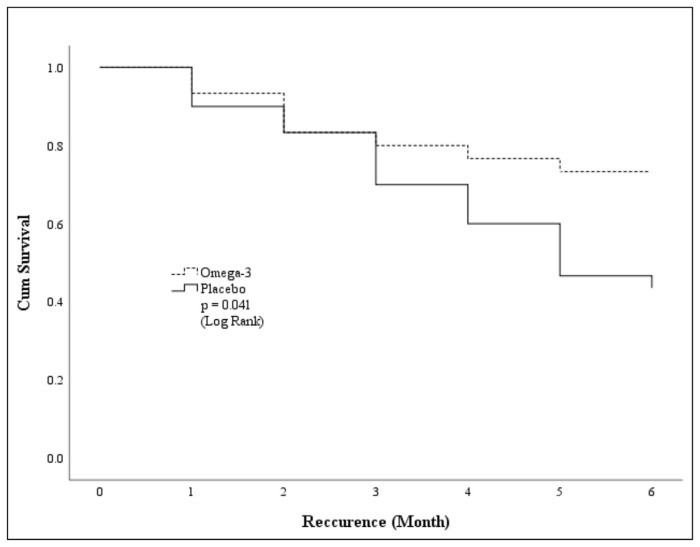
Survival rate comparison of recurrent MDD between the n-3 group and the placebo group.

Moreover, the 6-month survival rate of remitted MDD patients was higher in the n-3 group and statistically significant difference between groups ( *p*-value = 0.041) as shown in Fig. 2.

### 3.3. Comparison of erythrocyte PUFAs levels and other biochemical parameters between two groups at baseline and month 6 (endpoint)

Table 3 summarizes the changes in biochemical parameters at baseline and endpoint of intervention between groups. There were no significant differences of biochemical parameters such AST/GOT, ALT/GPT, BUN, creatinine, cholesterol, triglycerides, LDL, HDL, PT, aPTT, glucose, and prolactin between groups both at baseline and endpoint ( *p*-value> 0.05). In a comparison of AA, EPA, and DHA levels, there was no significant difference in AA level between the n-3 group and the placebo group at month 6 ( *p*-value = 0.141), but the EPA of the n-3 group after treatment was significantly higher than that of the control group ( *p-*value = 0.023), and the DHA in the n-3 group after treatment tended to be higher than those in the placebo but did not reach a significant difference ( *p-*value = 0.119).

## 4. Discussion

### 4.1. Main findings

To the best of our knowledge, this is the first study assessing the prophylactic effect of n-3 PUFAs monotherapy in preventing the recurrent events in remitted MDD patients for 6 months follow up. The results highlighted the potential of n-3 PUFAs monotherapy as a prophylactic treatment option for individuals with a history of MDD. The primary results of this study revealed a significant reduction in the recurrence of depressive episodes among participants receiving EPA-predominant n-3 PUFAs monotherapy with better survival rate at month 6 compared to the placebo. EPA-predominant n-3 PUFAs supplementation has been extensively studied and is believed to contribute to therapeutic effects in MDD [19,28]. Therefore, EPA-predominant n-3 PUFAs might protect against recurrent depressive episodes and mitigate inflammation-induced mood dysregulation by addressing inflammation, a recognized factor in the development of depression [29]. In addition, the beneficial effects of n-3 PUFAs, specifically DHA, may also contribute to its prophylactic properties, as it has been shown to support neuronal function and promote synaptic plasticity [30] that could affect the improvement of depressive symptoms. To explore the potential antidepressant-like effects of n-3 PUFAs, examining specific biomarkers associated with depression progression could provide valuable molecular insights. For instance, biomarkers such as S100β, high mobility group box 1 (HMGB1), and NSE can be analyzed in human tissues, particularly plasma. N-3 PUFAs may modulate the release of these DAMPs by interfering with their binding to their receptors such as toll-like receptors (TLRs) and receptor for advanced glycation end product (RAGE), thereby mitigating the activation of inflammatory responses linked to depression [31]. Even though this study’s findings presented that monthly HRSD scores did not show any significant differences at each time point (month) between groups. However, HRSD scores were lower in the n-3 group compared to the placebo group in each month. It is plausible that the observed trend could be attributed to the limited size of the sample, which may have hindered the attainment of statistically significant outcomes.

### 4.2. Secondary outcomes

Secondary outcomes showed the biochemical parameters related to side effects of n-3 PUFAs monotherapy on function of organs such as liver, kidney, heart. Our study demonstrated that no significant adverse effects were caused. The use of n-3 PUFAs supplementation has been confirmed for its efficacy [16,32] and safety without adverse effects in treating depressive symptoms [33]. Furthermore, our study revealed increased plasma levels of EPA and DHA, with only EPA demonstrating statistical significance. However, this indicates that n-3 PUFAs monotherapy induced changes in erythrocyte PUFAs composition. The lack of significant changes in DHA levels may be attributed to its distinct metabolic role, compared to EPA. While EPA is rapidly utilized in anti-inflammatory processes and mood regulation, DHA primarily supports neuronal membrane structure, which may result in slower turnover [34]. Additionally, adequate baseline DHA levels could lead to a plateau effect with supplementation. These findings suggest that EPA may have a more immediate impact on mood, while DHA plays a complementary, long-term role. Future studies should investigate the delayed effects of DHA on neural health and its interactions with EPA in mood stabilization. Likewise, previous study found that 12-week n-3 PUFAs supplementation significantly contributed higher level of EPA in plasma but not with DHA [35]. On the other hand, clinical studies also reported that 8-week and 12-week n-3 PUFAs supplementation contributed to higher levels of both EPA and DHA in plasma [36]. These various findings might be affected by different lengths of intervention and different doses of n-3 PUFAs. Nevertheless, previous meta-analytic studies have emphasized that EPA has more beneficial effect on treating depression compared to DHA [19]. Overall, our study supported the idea that EPA-predominant n-3 PUFAs have a beneficial effect in preventing the risk of recurrent depressive episodes without any significant adverse effects. This study demonstrated that a 3-g dose of n-3 PUFAs could be deemed tolerable and effective in preventing MDD recurrence. This aligns with a previous meta-analysis study recommending a ≥2 g dose of n-3 PUFAs to reach beneficial reductions in mood scores [16]. Our study has several strengths. First, it employed a randomized controlled trial design with a 6-month intervention. Second, by focusing solely on n-3 PUFAs monotherapy, the study provides valuable insights into the monotherapy effects of n-3 PUFAs in preventing MDD recurrence. The clear research objective adds to the strength of the study, allowing for a focus on prophylactic effect of n-3 PUFAs. We acknowledge several limitations in our study. Primarily, dietary intake, specifically the incorporation of n-3 food resources, may have influenced the observed outcomes as well as the effects of medication history. Thus, future studies should take these relevant factors into account.

Moreover, side-effects related factors including emotional instability, cognitive impairments, behavioral changes, worsening of symptoms, psychosocial distress, or relapse due to inadequate treatment, placebo effects, or trial-related stress should be considered in the future study. While the role of circulating PUFAs and their metabolites was not explored in this work, we acknowledge their potential importance in modulating neural and behavioral responses. Future studies are warranted to investigate the relationship between PUFA-related metabolites, targeted neural parameters, and MDD to provide a more comprehensive understanding of the underlying mechanisms. For example, eicosanoids and resolvins, which play roles in inflammation and cell signaling in depression [37,38]. Furthermore, the study’s findings should be interpreted with caution due to the relatively modest sample size, notwithstanding attempts to address this limitation through the recruitment of participants from multiple centers. A key limitation of this study is the high attrition rate, which may have introduced bias by creating imbalances between the groups and reducing the generalizability of the findings. Despite efforts to address missing data, the potential for systematic differences between those who completed the study and those who dropped out could have impacted the reliability of the results.

Another limitation of this study is the lack of initial assessment of co-morbid physical diseases, which could influence the results. However, the study focused on the primary variables under investigation, and future research should incorporate these factors to control for potential confounders and improve generalizability. While a longer follow-up would be ideal to fully evaluate the long-term effects of n-3 PUFAs, the six-month period was selected to capture the initial and potentially significant impact of treatment on depression-related outcomes. Future studies with extended follow-up durations are needed to assess the long-term prophylactic effects, especially for relapsing conditions like MDD. Finally, the HRSD was employed not for diagnostic purposes but rather for assessing the severity of MDD.

### 4.3. Conclusion

In conclusion, the findings of this study provide evidence supporting the use of n-3 PUFAs, particularly EPA-predominant monotherapy, as a prophylactic measure against the recurrence of MDD and contributed to a better survival rate at month 6 of intervention. Incorporating n-3 PUFAs into the treatment regimen for individuals with a history of MDD could offer a promising approach to long-term management and prevention. Further study is warranted to elucidate the underlying mechanisms and optimize the use of n-3 PUFAs as a prophylactic intervention in preventing recurrence of MDD.

## Figures and Tables

**Table 1:** Characteristics of participants at baseline.

Characteristics	n-3 Group (n = 30)	Placebo (n = 30)	*p-*value^a^
Age, years	38.3 ± 12.9	39.3 ± 14.1	0.768
Male (n, %)^b^	(25, 83.3)	(25, 83.3)	0.994
Depression severity			
HRSD	6.0 ± 1.9	6.0 ± 2.0	0.947
BDI	13.7 ± 11.5	15.2 ± 11.2	0.618

Mean ± SD (all such values), n-3: Omega-3, HRSD: Hamilton Rating Scale for Depression, BDI: Beck Depression Inventory.

a Mann–Whitney U Test unless stated otherwise.

b Chi-square test.

**Table 2a:** Comparison of HRSD scores between two groups at each time point.

Assessment (Month)	HRSD Scores		*p*-value^a^

	n-3 Group	Placebo	
0	6.0 ± 1.9, 30	6.0 ± 2.0, 30	0.947
1	6.0 ± 3.3, 30	7.8 ± 4.9, 30	0.109
2	6.6 ± 3.4, 28	7.0 ± 4.6, 27	0.653
3	7.3 ± 4.0, 26	8.2 ± 6.3, 25	0.567
4	7.1 ± 4.9, 24	8.7 ± 6.3, 21	0.324
5	7.0 ± 5.5, 23	8.9 ± 7.0, 18	0.294
6	7.1 ± 5.7, 22	8.9 ± 6.8, 14	0.324

Mean ± SD, n (all such values), n-3: Omega-3.

a Mann–Whitney U Test.

**Table 2b:** The incidence of MDD recurrence between the two groups at each time point.

	Assessment (Month)						

	0	1	2	3	4	5	6
n-3 Group	0/30	0/30	2/30	4/30	6/30	7/30	8/30
Placebo	0/30	0/30	3/30	5/30	9/30	12/30	16/30
*p*-value^a^	–	–	0.640	0.718	0.371	0.165	0.035*

Data presented as incidence of recurrence/number of participants of each group,

* *p*-value <0.05,

n-3: Omega-3.

a Chi-square Test.

**Table 3:** Comparison of erythrocyte PUFAs and biochemical parameters levels between two groups at baseline and month 6 (endpoint).

Parameters	Baseline			Month 6		
	
	n-3 Group (n = 30)	Placebo (n = 30)	*p*-value^a^	n-3 Group (n = 22)	Placebo (n = 14)	*p*-value^a^
AST (GOT)	20.39 ± 6.774	21.04 ± 4.514	0.696	21.50 ± 6.587	23.90 ± 5.665	0.379
ALT (GPT)	19.00 ± 17.015	18.65 ± 7.504	0.926	18.12 ± 6.833	16.50 ± 3.171	0.512
BUN	13.12 ± 3.140	13.85 ± 3.416	0.426	14.00 ± 4.648	12.50 ± 2.747	0.352
Creatinine	0.90 ± 0.182	0.99 ± 0.659	0.528	0.90 ± 0.130	0.89 ± 0.202	0.810
Albumin	4.32 ± 0.291	4.35 ± 0.391	0.762	4.32 ± 0.324	4.33 ± 0.284	0.879
Cholesterol	172.00 ± 13.223	175.13 ± 17.254	0.578	164.86 ± 14.253	156.83 ± 57.464	0.726
Triglycerides	78.78 ± 39.788	91.29 ± 39.154	0.330	72.30 ± 40.000	95.90 ± 40.397	0.206
HDL	55.01 ± 13.819	58.50 ± 16.168	0.422	51.09 ± 13.794	57.60 ± 11.520	0.233
LDL	106.39 ± 14.826	103.89 ± 18.000	0.692	93.15 ± 11.728	120.51 ± 26.098	0.119
PT (secs)	11.50 ± 0.665	11.54 ± 0.565	0.836	11.54 ± 0.415	11.62 ± 0.545	0.529
APTT (secs)	30.38 ± 2.196	30.10 ± 2.767	0.717	31.54 ± 2.408	30.37 ± 2.157	0.720
Glucose	92.78 ± 5.954	91.89 ± 7.711	0.654	92.33 ± 5.990	95.91 ± 7.217	0.209
Prolactin	9.98 ± 8.518	10.46 ± 4.966	0.847	10.60 ± 4.916	12.36 ± 6.991	0.579
AA	5.38 ± 0.35	5.38 ± 0.22	0.989	5.83 ± 0.335	5.38 ± 0.284	0.141
EPA	0.80 ± 0.01	0.79 ± 0.02	0.945	0.85 ± 0.041	0.79 ± 0.025	0.023*
DHA	3.23 ± 0.05	3.27 ± 0.05	0.184	3.81 ± 0.660	3.30 ± 0.073	0.119

Mean ± SD (all such values),

* *p*-value <0.05,

n-3: omega-3, GOT: Glutamate Oxaloacetate transaminase, GPT: Glutamate Pyruvate transaminase, BUN: Blood Urea Nitrogen, HDL: High-density lipoprotein, LDL: Low-density lipoprotein, PT: Prothrombin time, APTT: Activated Partial Thromboplastin Time, AA: Arachidonic Acid, EPA: Eicosapentaenoic Acid, DHA: Docosahexaenoic Acid.

a Mann–Whitney U Test.

## Abbreviations

RCT: Randomized Controlled Trial
MDD: Major Depressive Disorder
DSM-IV: The Diagnostic and Statistical Manual of Mental Disorders, Fourth Edition
HRSD: Hamilton Rating Scale for Depression
BDI: Beck’s Depression Inventory
N-3 PUFAs: Omega-3 Polyunsaturated Fatty Acids
DHA: Docosahexaenoic Acid
EPA: Eicosapentaenoic Acid
AA: Arachidonic Acid
AST/GOT: Aminotransferase
ALT/GPT: Alanine Aminotransferase
BUN: Blood Urea Nitrogen
LDL: Low-density Lipoprotein
HDL: High-density Lipoprotein
PT: Prothrombin time
aPTT: Activated Partial Thromboplastin Time
FAME: Fatty Acid Methyl Esters
CVD: Cardiovascular Disease
DAMPs: Damage-Associated Molecular Patterns
HMGB1: High Mobility Group Box 1
NSE: Neuron Specific Enolase
TLR: Toll-Like Receptor
RAGE: Receptor for Advanced Glycation End Product
